# Cost‐effectiveness analysis of continuing bevacizumab plus chemotherapy versus chemotherapy alone after first progression of metastatic colorectal cancer

**DOI:** 10.1002/cam4.6904

**Published:** 2024-01-08

**Authors:** Yulian Li, Min Hu, Zhe Zhang, Mingming Chu, Rufu Xu, Lulu Liu, Wenxing Dong, Mengmeng Yang, Rong Zhang

**Affiliations:** ^1^ Department of Pharmacy The Second Affiliated Hospital of Army Medical University Chongqing People's Republic of China

**Keywords:** bevacizumab, biosimilar, colorectal neoplasms, cost‐effectiveness, drug therapy

## Abstract

**Background:**

Continuation of bevacizumab plus second‐line chemotherapy has significantly improved overall and progression‐free survival in patients with metastatic colorectal cancer (mCRC). However, the cost‐effectiveness of such high cost therapy is still uncertain in China; so this analysis was performed to evaluate the cost‐effectiveness of these treatment options from the Chinese health care system perspective.

**Methods:**

A cost‐effectiveness analysis was conducted using data from the ML18147 trial (ClinicalTrials.gov identifier NCT00700102) by modeling a partitioned survival model. Main evaluation indicators were quality‐adjusted life years (QALYs) and incremental cost‐effectiveness ratio (ICER) with a willingness to pay (WTP) threshold of $38,201 per QALY. One‐way and probabilistic sensitivity analyses were conducted to assess the robustness and stability of the model. Subgroup and scenario analyses were also performed to make our study more relevant.

**Results:**

Bevacizumab plus chemotherapy increased 0.12 QALYs and an incremental cost of $22,761.62 compared with chemotherapy, resulting in an ICER of $188,904.09 per QALY. The model was most sensitive to the utility of progression‐free survival and the cost of bevacizumab. Compared with chemotherapy, bevacizumab plus chemotherapy had a 0% cost‐effectiveness probability, and no cost‐effectiveness in subgroups at the WTP threshold of $38,201 per QALY. The scenario analysis found that bevacizumab biosimilar gained an ICER of $126,397.38 per QALY when assuming the cost of drugs was calculated at the most affordable price.

**Conclusions:**

At the WTP threshold of $38,201 per QALY, continuation of bevacizumab plus chemotherapy is unlikely considered cost‐effective for patients after first progression of mCRC.

## 1 INTRODUCTION

Colorectal cancer (CRC) is among the leading malignancies, ranked third in incidence and second in cancer mortality globally.^1^ In China, it is the second in incidence and fourth in mortality based on the latest national cancer statistical data issued by the Chinese National Cancer Center in February 2022.^2^ Additionally, up to 50% of CRC patients have distant metastases when diagnosed.^3^ Available evidence supports that survival of metastatic colorectal cancer (mCRC) has increased in progress with chemotherapy and targeted medicine.^4^

Bevacizumab (Avastin®; Roche Pharma [Schweiz] Ltd) is a recombinant humanized monoclonal antibody that inhibits the vascular endothelial growth factor (VEGF) to inhibit VEGF‐related endothelial cell proliferation and angiogenesis.^5^ It is the only targeted medicine that promotes survival when combined with chemotherapy in the first‐line treatment for mCRC, regardless of the mutational status of the gene and tumor location. Moreover, bevacizumab combined with chemotherapy has a significant benefit in overall survival (OS) and progression‐free survival (PFS) in second‐line treatment.^6^ The ML18147 is the first Phase 3 randomized controlled trial (ClinicalTrials.gov identifier NCT00700102) assessed the effect on continuing bevacizumab beyond progression of mCRC in patients who had previously been given bevacizumab plus standard first‐line chemotherapy, its primary end‐point was OS, and the secondary end‐point was PFS. The results showed that bevacizumab in combination with chemotherapy significantly prolonged the OS and PFS in comparison to chemotherapy as a second‐line treatment for mCRC patients (OS, 11.2 months vs. 9.8 months, hazard ratio [HR], 0.81, 95% CI 0.69–0.94; PFS, 5.7 months vs. 4.1 months, HR, 0.68, 95% CI 0.59–0.78).^7^ Based on its significant clinical efficacy in mCRC, the US National Comprehensive Cancer Network (NCCN) and the Chinese Society of Clinical Oncology (CSCO) guidelines recommend bevacizumab in combination with chemotherapy as second‐line treatment for mCRC.^8^, ^9^

Despite remarkable clinical efficacy, high costs of bevacizumab have imposed an enormous financial burden on patients and the national health care system.^10^ To our knowledge, only one literature collected data in 2013 has evaluated the cost‐effectiveness of bevacizumab in second‐line treatment of mCRC from the perspective of the US payers, the results suggested that adding bevacizumab to second‐line chemotherapy was not affordable.^11^ In China, the price of bevacizumab has reduced by 72% over the past decade due to the reform of medical insurance system and massive upsurge of biosimilars with the expiration of bevacizumab.^12^ Moreover, the price of bevacizumab is likely to further reduce for Chinese centralized procurement in the future.^13^ In recent years, several studies showed that bevacizumab in combination with chemotherapy was not cost‐effective as first‐line treatment for mCRC patients from the perspective of the Chinese healthcare system.^14^, ^15^, ^16^, ^17^ However, no such cost‐effectiveness analyses have been studied as a second‐line therapy.

Considering that cost‐effectiveness analyses of bevacizumab are helpful to optimally distribute limited health care resources for clinicians and decision makers, we conducted this analysis based on the results of the ML18147 trial to appraise the cost‐effectiveness of continuing bevacizumab combined with chemotherapy in comparison to chemotherapy as a second‐line therapy for mCRC from the Chinese health care system perspective, which can offer reference for clinical treatment option and medical decision‐making in China.

## 2 MATERIALS AND METHODS

### 2.1 Patient population

This study adhered to the economic evaluation.^18^ The evaluation was based on a literature review of publicly available data and modeling techniques, which did not require Institutional Review Board (IRB) review or exemption by the Ethics Committee. The population of patients targeted was the same as the ML18147 trial. Patients who qualified for this analysis primarily included histologically confirmed mCRC and have progressed after treating with bevacizumab plus first‐line chemotherapy. Patients were randomly assigned to bevacizumab plus chemotherapy or chemotherapy alone. Chemotherapy regimens included fluorouracil (FU) plus oxaliplatin (FOLFOX) or irinotecan (FOLFIRI). The two chemotherapy regimens can be alternatively as first and second‐line treatment because of similar efficacy in mCRC (i.e., chemotherapy in patients who were given first‐line oxaliplatin was switched with second‐line irinotecan and vice versa). The median age of the patients were 63 years, and the percentage of male in the bevacizumab plus chemotherapy group was 65% compared to 63% in the chemotherapy group.^7^ Detailed patient characteristics are shown in Table S1.

### 2.2 Model construction and clinical data

**FIGURE 1:**
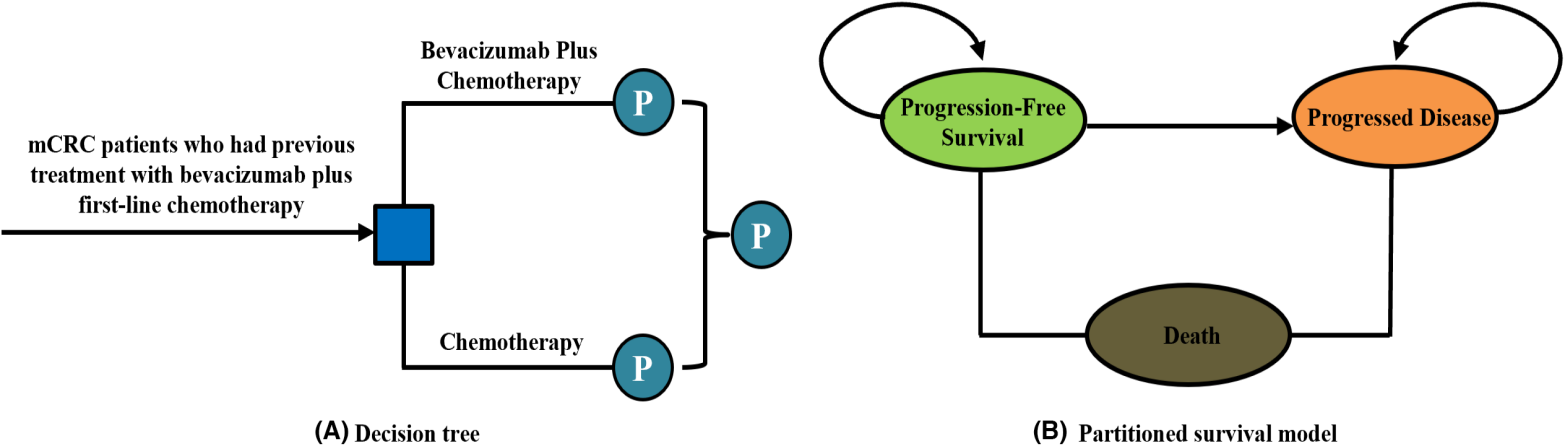
Partitioned survival model.

A partitioned survival model (PSM) was constructed to appraise the cost‐effectiveness of economic evaluation from the Chinese health care system perspective (see Figure 1). The model has three health states, including PFS, progressive disease (PD), and death. These three health states were mutually exclusive and allocated health utility score individually. All patients entered the model with PFS, a certain proportion of patients would enter PD after disease progression, and each health state had a certain probability of transferring to death, and all transition proportion between states were derived from the ML18147 trial.

To determine whether second‐line bevacizumab plus chemotherapy is more cost‐effective than chemotherapy alone for mCRC, the PSM model generated an incremental cost‐effectiveness ratio (ICER) between the two competing regimens with a two‐week cycle. ICER compared the healthcare costs per additional quality‐adjusted life year (QALY) with the willingness‐to‐pay (WTP) threshold, which was set at three times Chinese per capita GDP in 2022.^19^ The time horizon was 4 years because all patients died at this time point. The PSM model was programmed in TreeAge Pro version 2022 (TreeAge Software LLC, Williamstown, Massachusetts).

Proportion of patients in PFS and OS in the bevacizumab plus chemotherapy arm and the chemotherapy arm were derived from the Kaplan–Meier curves by GetData Graph Digitizer.^20^ The proportion of patients in PD was calculated by the difference between the OS and PFS curves, mortality was calculated by subtracting the probability of OS from one. Because all patients in ML18147 trial were followed up to death so that the Kaplan–Meier curves were whole, it was not needed to extrapolate the survival curve.

### 2.3 Cost and utility

Only direct health care costs were considered (see Table 1) because this analysis was from the perspective of the Chinese health care system. All costs were converted to US dollars at the 2022 exchange rate: $1 = ¥6.73, and were calibrated to 2022 value using the consumer price index.^19^ Because quality‐of‐life data were not reported in the ML18147 study, we used it from patients with mCRC in Japan, which assigned quality‐of‐life utilities to patients who progressed before (0.78) and after (0.66).^21^

**TABLE 1:** Key model inputs.

Parameter	Value (range)	Distribution	Data source
Clinical input			
Proportion of receiving irinotecan‐based chemotherapy treatment in B + C regimen	0.41 (0.31–0.51)	Beta	Bennouna^7^
Proportion of receiving irinotecan‐based chemotherapy treatment in C regimen	0.42 (0.32–0.53)	Beta	Bennouna^7^
Proportion of receiving oxaliplatin‐based chemotherapy treatment in B + C regimen	0.59 (0.44–0.74)	Beta	Bennouna^7^
Proportion of receiving oxaliplatin‐based chemotherapy treatment in C regimen	0.58 (0.44–0.73)	Beta	Bennouna^7^
Weight per patient (kg)	64.30 (48.23–80.38)	Triangular	Chinese Bureau^22^
Body surface area (m^2^)	1.66 (1.24–2.07)	Triangular	Chinese Bureau^22^
Drug costs, $			
Bevacizumab per 100 mg	222.88 (167.16–278.60)	Gamma	Chinese drug^12^
Irinotecan per 40 mg	6.30 (4.73–7.88)	Gamma	Chinese drug^12^
Oxaliplatin per 50 mg	39.94 (29.96–49.93)	Gamma	Chinese drug^12^
5‐fluorouracil per 250 mg	10.03 (7.52–12.54)	Gamma	Chinese drug^12^
Folinate per 300 mg	3.66 (2.75–4.58)	Gamma	Chinese drug^12^
Fruquintinib per 5 mg	68.22 (51.17–85.28)	Gamma	Chinese drug^12^
Grade 3–5 AEs cost, $			
Neutropenia	161.24 (120.93–201.55)	Gamma	Wu^23^
Leucopenia	161.23 (120.92–201.54)	Gamma	Wu^23^
Diarrhea	14.96 (11.22–18.70)	Gamma	Wu^23^
Vomiting	78.50 (58.88–98.13)	Gamma	Wu^23^
Nausea	78.50 (58.88–98.13)	Gamma	Wu^23^
Venous thromboembolic	3412.78 (2559.59–4265.98)	Gamma	Goldstein^11^, ^12^
Follow‐up and surveillance costs, $			
Registration	2.93 (2.20–3.66)	Gamma	AOFC^24^, ^25^, ^26^, ^27^, ^28^
Laboratory tests	160.26 (121.20–200.33)	Gamma	Han^15^
Computed tomography	220.69 (165.52–275.86)	Gamma	AOFC^24^, ^25^, ^26^, ^27^, ^28^
Health utilities			
PFS health state	0.78 (0.59–0.98)	Beta	Kashiwa^21^
PD health state	0.66 (0.56–0.66)	Beta	Kashiwa^21^
Subsequent treatment			
Probability of receiving fruquintinib	0.55 (0.41–0.69)	Beta	Kashiwa^21^
Fruquintinib per 5 mg, $	68.22 (51.17–85.28)	Gamma	Chinese drug^12^
Hospice care			
Probability of receiving hospice care, %	0.30 (0.23–0.38)	Beta	Yao^29^
Hospice care costs per patient, $	873.41 (655.06–1091.76)	Gamma	Liu^30^
Hospitalization per cycle	58.50 (43.88–73.13)	Gamma	Wu^23^
Peripherally inserted central catheter (PICC)	24.00 (18.00–30.00)	Gamma	Wu^23^

Abbreviations: AE, adverse event; AOFC, average of Chinese five representative cities; B + C, bevacizumab plus chemotherapy; C, chemotherapy; PD, progressive disease; PFS, progression‐free survival.

According to the ML18147 trial, chemotherapy regimens included FOLFOX and FOLFIRI: leucovorin 400 mg/m^2^, 5‐fluorouracil 400 mg/m^2^ intravenously bolus on Day 1, followed by 2400 mg/m^2^ over 46–48 h intravenously continuous infusion, and oxaliplatin 85 mg/m^2^ (FOLFOX chemotherapy) or irinotecan 180 mg/m^2^ (FOLFIRI chemotherapy). Bevacizumab was given intravenously at a dosage of 5 mg/kg every 2 weeks with patients (59%) receiving FOLFOX chemotherapy or patients (41%) receiving FOLFIRI chemotherapy, while in the chemotherapy regimen, patients (58%) received FOLFOX and patients (42%) received FOLFIRI.^7^ We hypothesized the body surface area was 1.66 m^2^ and weight was 64.3 kg for the mean value according to a report among Chinese residents to calculate the dosage of drugs.^22^ When the disease progressed, 55% of patients were expected to receive fruquintinib as a subsequent therapy.^21^ All costs of drugs were derived from the average value of Chinese health industry data platform.^12^

Management of three to five adverse events (AEs) were considered to be incurred during the first cycle from published literature (see Table S2), and the costs of AEs were also considered.^11^, ^23^ Costs of follow‐up included registration fees, laboratory tests, computed tomography, which were derived from published literature and mean value of health care services in five Chinese cities including Shanghai, Beijing, Chongqing, Changsha, and Shenyang.^15^, ^24^, ^25^, ^26^, ^27^, ^28^ In addition, according to two studies based on Chinese populations, 0.30% patients received hospice care before death.^29^, ^30^

### 2.4 Base‐case analysis

ICER is expressed as the healthcare costs per additional QALY gained, which compares the cost of bevacizumab plus chemotherapy to the cost of chemotherapy alone. The recommendation was to assume cost‐effectiveness when the ICER was below the WTP threshold.^31^ Costs and QALYs were discounted at an annual rate of 5%.^31^ Additionally, using the following formulas, we calculated the incremental net health benefit (INHB) and incremental net monetary benefit (INMB): INHB(*λ*) = (μ_E1_ − μ_E0_) − (μ_C1_ − μ_C0_)/*λ* = ΔE − ΔC/*λ* and INMB(*λ*) = (μ_E1_ − μ_E0_) × *λ* − (μ_C1_ − μ_C0_) = ΔE × *λ* − ΔC, where μ_Ci_ and μ_Ei_ were the cost and effectiveness of bevacizumab plus chemotherapy (*i* = 1) or chemotherapy (*i* = 0), and *λ* was the WTP threshold.^32^, ^33^

### 2.5 Sensitivity analysis

We performed one‐way sensitivity analyses and probabilistic sensitivity analysis (PSA) to assess the robustness and stability of the model results. Parameters included in the one‐way sensitivity analyses were varied by 95% CIs as described in the literature or a variation of 25% from the base parameters were assumed to determine which had the greatest effects on ICER (see Table 1). To determine the impact of model uncertainty, 10,000 Monte‐Carlo simulations with randomly selected samples from the distributions of model inputs were performed simultaneously while changing all parameters.

### 2.6 Subgroup and scenario analyses

Weibull parametric hazard function was used to explore the uncertainty of economic outcomes in prespecified subpopulations derived from ML18147 trial, because it can have an incidence of increased hazards and is suitable for modeling the events happening early in follow‐up periods. In the subgroup analyses, the ICER was calculated for different patient characteristics by varying the HRs for OS. In addition, based on the Chinese situation that both bevacizumab biosimilars and chemotherapy drugs are produced by many different pharmaceutical companies, we conducted a scenario analysis to assume the cost of drugs was calculated at the most affordable price, and bevacizumab biosimilar have similar efficacy as its reference product with the lowest price.

## 3 RESULTS

### 3.1 Base‐case analysis

In base‐case analysis, bevacizumab combined with chemotherapy was linked to an increase of 0.12 QALYs (0.16 overall life‐years) and an incremental cost of $22,761.62 in comparison to second‐line chemotherapy, resulting an ICER of $188,904.09 per QALY. Furthermore, the INHB and INMB of bevacizumab combined with chemotherapy were −0.48 QALYs and $−18,177.50 (see Table 2).

**TABLE 2:** Summary of cost and outcome results in the base‐case analysis.

Strategy	Chemotherapy	Bevacizumab plus chemotherapy
Cost, $	8027.41	30,789.03
Life‐years	4 (3–5)	1.18
QALYs	0.73	0.85
ICER	NA	188,904.09
INHB^a^, QALY	NA	−0.4758
INMB^a^, $	NA	−18,177.50

Abbreviations: ICER, incremental cost‐effectiveness ratio; INHB, incremental net health benefit; INMB, incremental monetary benefit; QALYs, quality‐adjusted life‐years.

^a^ Compared with chemotherapy.

### 3.2 Sensitivity analysis

**FIGURE 2:**
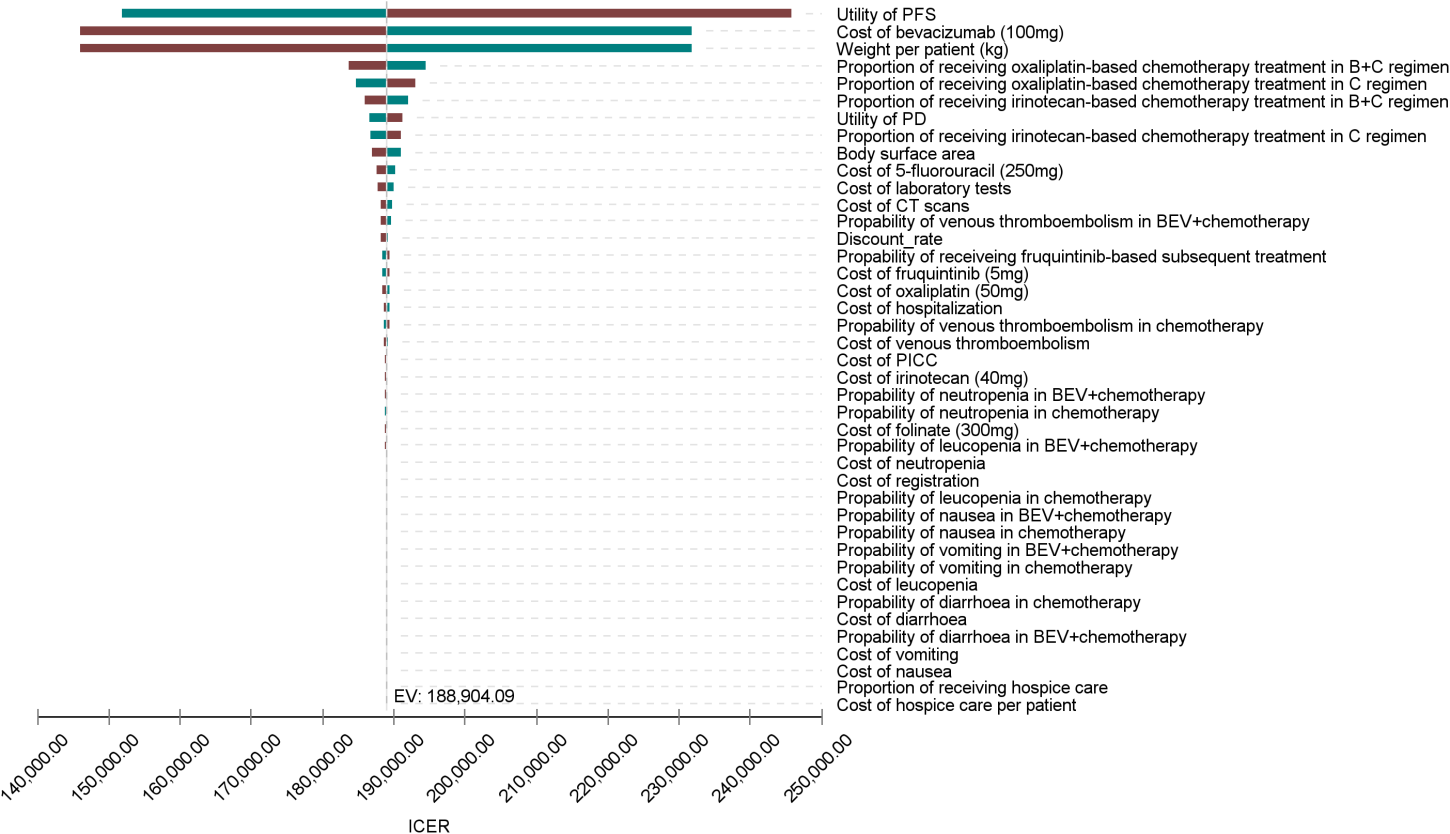
Tornado diagram for one‐way sensitivity analyses in the base case.

One‐way sensitivity analyses revealed that the model was most sensitive to utility of PFS, the cost of bevacizumab, and weight of patients (see Figure 2). When the upper limit of the utility for PFS was adopted (utility = 0.98), the ICER for bevacizumab plus chemotherapy versus chemotherapy was $151,896.25 per QALY, and when the lower limit (utility = 0.59) was adopted, the ICER was $245,795.08 per QALY. When the cost of bevacizumab was discounted by 25%, the ICER was $145,988.54 per additional QALY. The high body weight was associated with ICER over the threshold of $38,201/QALY. The remaining parameters, such as the proportion of receiving chemotherapy treatment, utility of PD, and body surface area had only modest or low correlation with the ICER. One‐way sensitivity analysis indicated that bevacizumab plus chemotherapy was not cost‐effective compared with chemotherapy at the lower or upper limits of any variable examined, which demonstrated the robustness of our findings. To attain more advantageous cost‐effectiveness (see Figure S1), the price of bevacizumab would need to be reduced from $222.88 to $87.49 (a reduction of 60.75%).

In the PSA of 10,000 simulations, the ICER scatterplot (see Figure S2) and the cost‐effectiveness acceptability curve (see Figure S3) indicated that bevacizumab combined with chemotherapy was not cost‐effective at the WTP threshold of $38,201. Furthermore, the scatterplot showed that all points were over the WTP, which indicated that ICER was always above the WTP when all parameters were varied within the range of values simultaneously, proving the robustness of our results. Moreover, the probability of bevacizumab combined with chemotherapy being cost‐effective was 50.00% when the WTP was $186,584.12, but there was no possibility that bevacizumab combined with chemotherapy would be cost‐effective when the WTP was $38,201 according to the cost effectiveness acceptability curve.

### 3.3 Subgroup and scenario analyses

Subgroup analyses showed that the ICER for bevacizumab plus chemotherapy versus chemotherapy was higher than $38,201 per QALY for all patients in the relevant clinical subgroups (see Table 3). The minimum value of ICER was calculated as $131,692.53 in male patients and the subgroup with first‐line progression‐free survival beyond 9 months. For all subgroups, bevacizumab plus chemotherapy was associated with negative INHBs at the WTP threshold of $38,201 by varying the HRs for OS. When the cost of drugs was calculated at the most affordable price, the overall cost of bevacizumab plus chemotherapy was $15,230.00, resulting in an ICER of $126,397.38 per QALY, and bevacizumab biosimilar would need to be reduced from $149.78 to $113.40 (a reduction of 24.29%) to be cost‐effective (see Figure S4).

**TABLE 3:** Summary of subgroup analyses obtained by varying the HRs for overall survival.

Subgroups	HR of OS^a^	Change in cost, $^b^	Change in QALYs^b^	ICER per QALY, $	INHB per QALY^b^	INMB^b^
All	0.81 (0.69–0.94)	22,773.98	0.118519	192,153.94	−0.4776	−18,246.44
Sex						
Female	0.99 (0.77–1.28)	22,714.33	0.022151	1,025,417.63	−0.5724	−21,868.14
Male	0.73 (0.60–0.88)	22,800.19	0.173132	131,692.53	−0.4237	−16,186.37
Age (years)						
<65	0.79 (0.65–0.98)	22,780.51	0.131357	173,423.82	−0.4650	−17,762.54
≥65	0.83 (0.66–1.04)	22,767.47	0.106173	214,436.96	−0.4898	−18,711.56
ECOG performance status						
0	0.74 (0.59–0.94)	22,796.90	0.165805	137,491.94	−0.4310	−16,462.98
≥1	0.87 (0.71–1.06)	22,754.47	0.082847	274,655.43	−0.5128	−19,589.63
First‐line progression‐free survival (months)						
≤9	0.89 (0.73–1.09)	22,748.00	0.071818	316,745.32	−0.5237	−20004.48
>9	0.73 (0.58–0.92)	22,800.19	0.173132	131,692.53	−0.4237	−16186.37
First‐line chemotherapy						
Oxaliplatin based	0.79 (0.62–1.00)	22,780.51	0.131357	173,423.82	−0.4650	−17762.54
Irinotecan based	0.82 (0.67–1.00)	22,770.72	0.112287	202,791.00	−0.4838	−18481.24
Time since last bevacizumab (days)						
≤42	0.82 (0.69–0.97)	22,770.72	0.112287	202,791.00	−0.4838	−18481.24
>42	0.76 (0.55–1.06)	22,790.33	0.151602	150,329.64	−0.4450	−16998.98
Liver metastasis only						
No	0.81 (0.67–0.97)	22,773.98	0.118519	192,153.94	−0.4776	−18246.44
Yes	0.79 (0.59–1.05)	22,780.51	0.131357	173,423.82	−0.4650	−17762.54
Number of organs with metastasis						
1	0.83 (0.64–1.08)	22,767.47	0.106173	214,436.96	−0.4898	−18711.56
>1	0.77 (0.64–0.94)	22,787.06	0.144717	157,459.36	−0.4518	−17258.73

^a^ The HR of overall survival represents the HR of bevacizumab plus chemotherapy versus chemotherapy for overall survival.

^b^ The results of bevacizumab plus chemotherapy minus chemotherapy.

Abbreviations: HR, hazard ratio; ICER, incremental cost‐effectiveness ratio; INHB, incremental net health benefit; INMB, incremental net monetary benefit; OS, overall survival; QALYs, quality‐adjusted life‐years.

## 4 DISCUSSION

Chemotherapy with or without bevacizumab has been broadly adopted for mCRC, contrast to the first‐line treatment, bevacizumab is not limited by tumor location in the second‐line therapy.^8^, ^9^ Several studies have been investigated to determine the cost‐effectiveness of bevacizumab in combination with chemotherapy as first‐line treatment for patients with mCRC from the perspective of the Chinese healthcare system.^14^, ^15^, ^16^, ^17^ Zhang et al. found that bevacizumab plus capecitabine was not a cost‐effective treatment option for elderly patients with previously untreated mCRC.^17^ Wen et al. reported that RAS screening before bevacizumab therapy seemed as the most cost‐effective strategy with the shortest QALYs, compared with cetuximab (a monoclonal antibody of epidermal growth factor receptor, anti‐EGFR mAb).^14^ Han et al. reported that bevacizumab plus FOLFIRI was not more cost‐effective than cetuximab plus FOLFIRI in Chinese patients with left‐sided RAS WT mCRC.^15^ Lee et al. reported that compared with bevacizumab, addition of anti‐EGFR mAb to chemotherapy was more cost‐effective in patients with pan‐RAS wild‐type (WT) and left‐sided pan‐RAS WT, especially in left‐sided pan‐RAS WT mCRC populations from the Chinese Hong Kong societal perspective.^16^

ML18147 trail reported significant survival benefits of continuing bevacizumab plus chemotherapy after first progression in mCRC patients previously receiving bevacizumab plus first‐line chemotherapy.^7^ Goldstein et al. determined that compared with FOLFIRI alone, bevacizumab plus FOLFIRI was not cost‐effective for the US payer in patients with mCRC who had progressed after receiving bevacizumab plus FOLFOX in first‐line therapy. However, no such cost‐effectiveness analyses have been studied in China. Considering high costs of bevacizumab have imposed a considerable financial burden on the Chinese health care system, and cost‐effectiveness analyses can offer reference for clinical treatment option and decision makers, we conducted the first study to examine the cost‐effectiveness of continuing bevacizumab to chemotherapy as second‐line therapy in mCRC from Chinese health care system. It is important to indicate several differences between our study and Goldstein et al.^11^ First, based on the completeness of the survival curve, we estimated the transition proportion for each state by extracting the points on the Kaplan–Meier curves directly instead of using the parametric survival functions. Second, despite FOLFOX and FOLFIRI having similar efficacy in such situations, differences in drug costs may influence the findings by fluctuating ICER, so we calculated the drug cost as the proportion of each chemotherapy regimen in the treatment arm of the original study rather than assuming FOLFIRI as second‐line chemotherapy. Finally, it may be attributed to consumption of different resource in different countries, our research showed that continuing bevacizumab plus chemotherapy after first progression would add 0.12 QALYs compared to second‐line chemotherapy at an incremental cost of US$22,761.62, resulting in an ICER of $188,904.09 per QALY in China, while the ICER for bevacizumab plus FOLFIRI was $364,083 per QALY compared to FOLFIRI from the perspective of US payers according to Goldstein et al. Similarly, both our findings indicated that continuing bevacizumab to second‐line chemotherapy was not a cost‐effective option for patients with mCRC.

The cost of bevacizumab was a primary element associated with economic outcomes apart from the utility of PFS, it would have to be reduced from $222.88 to $87.49 (a 60.75% reduction) to become cost‐effective compared with chemotherapy. The expiration of bevacizumab patents has allowed the development of bevacizumab biosimilars, and 10 were approved by NMPA at present.^34^ So we conducted a scenario analysis assuming that the cost of all drugs was calculated at the most affordable price, and bevacizumab biosimilars have similar efficacy as its reference product with the lowest price. Results showed that bevacizumab biosimilar had no cost‐effectiveness unless it decreased from $149.78 to $113.40 (a 24.29% reduction), which is consistent with Giuliani et al.^35^ In recent years, as the financial burden of cancer continues to increase, drug negotiations have become an essential means of reducing drug prices to ensure the sustainability of treatment for patients.^36^ The Chinese government has increasingly attached importance to the development of pharmacoeconomics in drug negotiations because it can provide valuable references for the reform of medical insurance system and centralized procurement.^13^ Additionally, Chinese government is gradually incorporating biological products into centralized procurement, and insulin is the first step in this exploration with an average price reduction of 48%, moreover, biosimilars are also being considered for inclusion in the centralized procurement, and bevacizumab may also be included. In addition, submission of applications for generic drug consistency evaluation were continued to the NMPA by pharmaceutical companies, which may lead to further cost reductions for bevacizumab and its biosimilars.^37^, ^38^ Our findings can therefore provide concrete economic evidence for policy development.

The capability of bevacizumab plus chemotherapy to protect against disease‐related deaths is a key correlate of economic results. According to the results of the subgroup analyses, bevacizumab plus chemotherapy was more cost‐effective for patients with an excellent prognosis than for patients with a bad prognosis, such as male patients or patients with first‐line progression‐free survival beyond 9 months. However, bevacizumab plus chemotherapy may not cost‐effectiveness for patients with a negative HR for OS who had a high risk of mortality, such as female patients or the subgroup with ECOG performance status equal to or greater than one, or first‐line progression‐free survival less than or equal to 9 months. Further work is needed to identify which patients with mCRC are most likely to benefit from continuing bevacizumab with chemotherapy after first progression.

There are several strengths of this study that need to be emphasized. First, to our knowledge, it is the first analysis to investigate the cost‐effectiveness of continuing bevacizumab to chemotherapy as second‐line treatment in mCRC from Chinese health care system. Bevacizumab has become a routine anti‐angiogenic target drug in the second‐line treatment of mCRC. However, there are no studies on the economics of bevacizumab in the second‐line treatment of mCRC in China, and our research suggested that policy makers can improve health benefits by allocating more resources to more affordable forms of intervention. Second, our analysis investigated the economic results of 16 prespecified subgroups in the ML18147 trial, it may help physicians, patients and decision‐makers to develop more precise individual treatments. Third, based on the enormous bevacizumab biosimilar market in China, our study investigated the cost‐effectiveness of bevacizumab biosimilars at the most affordable price for all drugs to provide a comprehensive economic assessment of bevacizumab. We believe the results are positive because of the continued submission of applications for generic drug consistency evaluation to the NMPA by pharmaceutical companies, which may make bevacizumab more cost‐effective.

There are also several limitations in the analysis. First, ML18147 trial is a clinical trial based on the Caucasian population, variability between nations and races may result in variable outcomes, including therapeutic efficacy, and adverse events (AEs). Although Cao et al. conducted a Phase II clinical study based on Chinese populations, the study included too few patients and the survival data were not sufficiently mature to build an economic model.^39^ Second, ML18147 trial has strict inclusion and exclusion criteria for patients, for example, patients need to have been continuously treated with first‐line bevacizumab more than 3 months, and patients would be excluded if first‐line progression‐free survival was less than 3 months. Strict inclusion and exclusion criteria for the trial, as well as racial differences, may influence economic outcomes by affecting OS and PFS. Therefore, our current analysis needs to be further validated by real‐world data. Third, the costs associated with Grade 1 and 2 AEs were not taken into account, which may have resulted in an overestimation of the economic impacts of bevacizumab plus chemotherapy. This constraint might not be a serious issue due to the low costs associated with AEs, as demonstrated in the one‐way sensitivity analysis. However, the incidence of AEs may be helpful information for physicians and policy makers.

In conclusion, continuing bevacizumab with chemotherapy after first progression was not cost‐effective for patients with mCRC from the perspective of Chinese health care system at a WTP threshold of $38,201 per QALY. The economic results may be maximized by adopting personalized therapy. Notably, bevacizumab biosimilars, as alternatives to original bevacizumab with lower prices, it may make continuing bevacizumab with chemotherapy more cost‐effective for patients with mCRC after first progression when it was discounted by 24.29%.

## AUTHOR CONTRIBUTIONS

**Yulian Li:** Conceptualization (equal); data curation (equal); formal analysis (equal); investigation (equal); resources (equal); software (equal); validation (equal); visualization (equal); writing – original draft (equal); writing – review and editing (equal). **Min Hu:** Conceptualization (equal); data curation (equal); formal analysis (equal); investigation (equal); methodology (equal); resources (equal); software (equal); validation (equal); visualization (equal); writing – original draft (equal); writing – review and editing (equal). **Zhe Zhang:** Conceptualization (equal); data curation (equal); writing – review and editing (equal). **Mingming Chu:** Data curation (equal); software (equal); writing – review and editing (equal). **Rufu Xu:** Data curation (equal); writing – review and editing (equal). **Lulu Liu:** Software (equal). **Wenxing Dong:** Software (equal). **Mengmeng Yang:** Conceptualization (equal); funding acquisition (equal); project administration (equal); supervision (equal); writing – review and editing (equal). **Rong Zhang:** Conceptualization (equal); funding acquisition (equal); project administration (equal); supervision (equal); writing – review and editing (equal).

## FUNDING INFORMATION

This work was supported by the Science Health Joint Medical Scientific Research Project of 345 Chongqing (2022MSXM011), People's Republic of China.

## CONFLICT OF INTEREST STATEMENT

The authors declare that the research was conducted in the absence of any commercial or financial relationships that could be construed as a potential conflict of interest.

## Supporting information


Data S1.

## Data Availability

The original contributions presented in the study are included in the article/supplementary material, further inquiries can be directed to the corresponding author/s.
