# Supplementary material for: Cost‐effectiveness analysis of continuing bevacizumab plus chemotherapy versus chemotherapy alone after first progression of metastatic colorectal cancer
Source: Cancer Med. 2024 Jan 8;13(1):e6904. doi: 10.1002/cam4.6904 (PMC10807607; doi:10.1002/cam4.6904)
Supplement: Supplementary file 1 — Data S1. [file CAM4-13-e6904-s001.docx]

**Table S1. Characteristics of patients at baseline.**

| **Characteristics** | | **Bevacizumab and**  **chemotherapy (n=409)** | **Chemotherapy alone (n=411)** |
| --- | --- | --- | --- |
| Sex | |  |  |
|  | Male | 267 (65%) | 259 (63%) |
|  | Female | 142 (35%) | 152 (37%) |
| Age (years) | | 63 (27-84) | 63 (21-84) |
| ECOG performance status | |  |  |
|  | 0 | 179 (44%) | 178 (43%) |
|  | 1 | 209 (51%) | 212 (52%) |
|  | 2 | 19 (5%) | 19 (5%) |
| First-line progression-free survival (months) | |  |  |
|  | ≤9 | 221 (54%) | 229 (56%) |
|  | >9 | 187 (46%) | 182 (44%) |
| Liver metastasis only | |  |  |
|  | No | 300 (73%) | 292 (71%) |
|  | Yes | 109 (27%) | 118 (29%) |
| Number of organs with metastases | |  |  |
|  | ≤1 | 148 (36%) | 160 (39%) |
|  | >1 | 261 (64%) | 250 (61%) |
| Time from last bevacizumab dose (days) | |  |  |
|  | ≤42 | 315 (77%) | 316 (77%) |
|  | >42 | 94 (23%) | 95 (23%) |
| First-line chemotherapy | |  |  |
|  | Irinotecan-based | 240 (59%) | 237 (58%) |
|  | Oxaliplatin-based | 169 (41%) | 174 (42%) |

Abbreviations: data are number (%) or median (range). ECOG=Eastern Cooperative Oncology Group.

**Table S2.** Incidence rate of adverse events (≥3 grade).

| **3-5 AEs** | **Incidence Rate of Bevacizumab Plus Chemotherapy, %** | **Incidence Rate of Chemotherapy, %** |
| --- | --- | --- |
| Neutropenia | 16 (12-20) | 13 (9.75-16.25) |
| Leucopenia | 4 (3-5) | 3 (2.25-3.75) |
| Diarrhoea | 10 (0.75-1.25) | 8 (6-10) |
| Vomiting | 3 (2.25-3.75) | 3 (2.25-3.75) |
| Nausea | 3 (2.25-3.75) | 3 (2.25-3.75) |
| Venous thromboembolism | 5 (3.75-6.25) | 3 (2.25-3.75) |


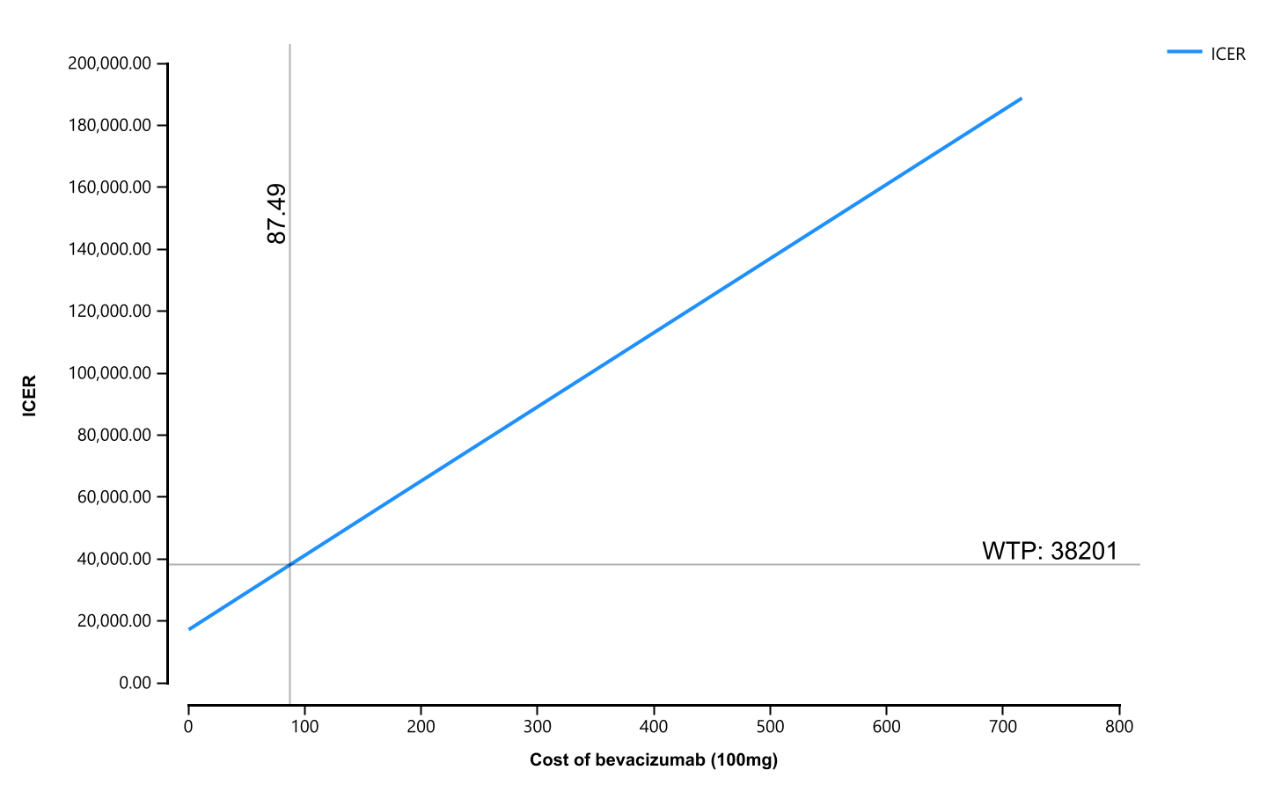


Figure S1. One‐way sensitivity analyses for cost of bevacizumab in base-case analysis


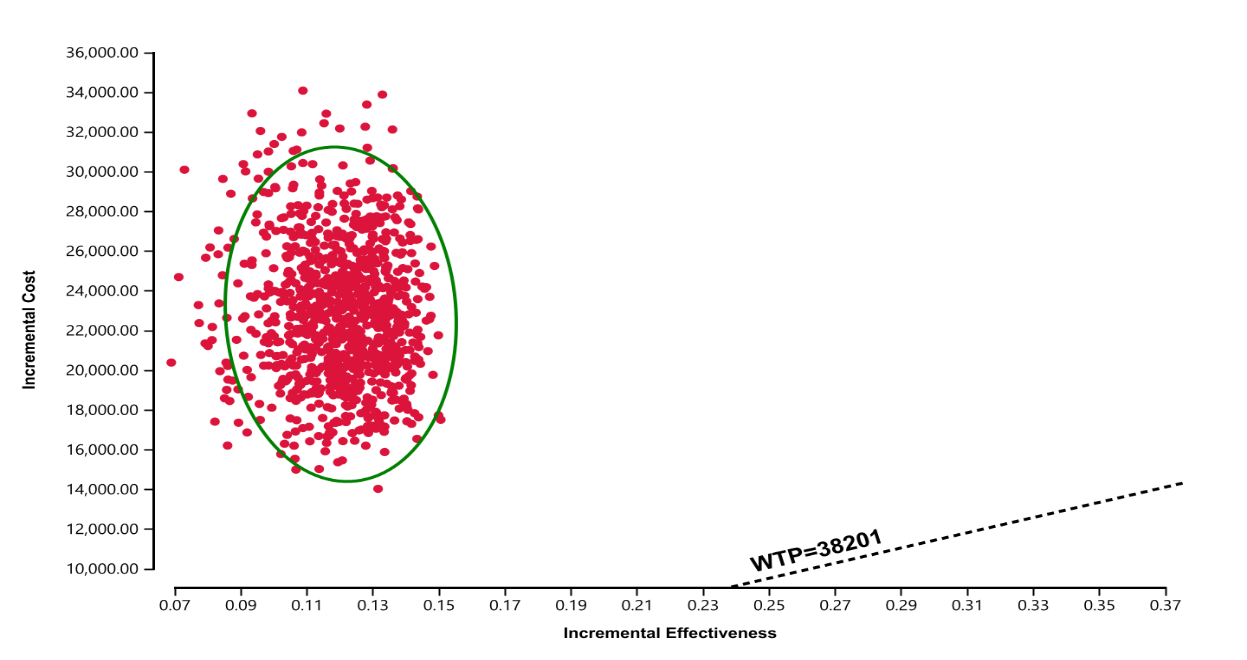


Figure S2. Scatter plot of probabilistic sensitivity analysis


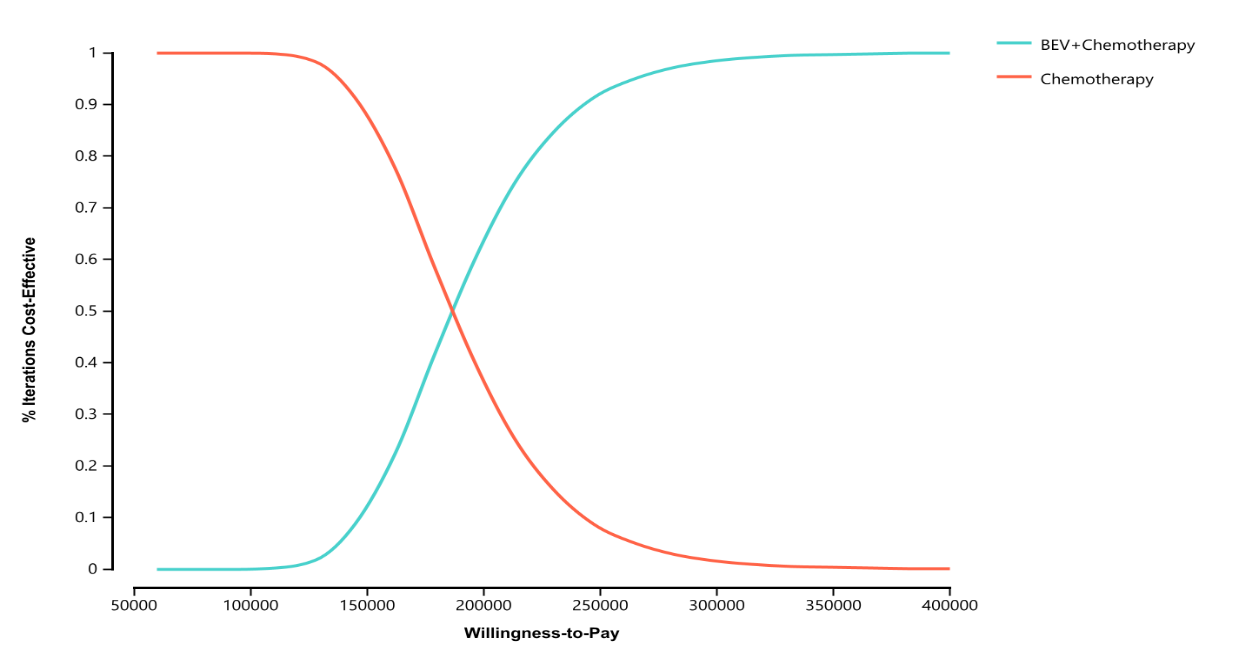


Figure S3. Acceptability curves for bevacizumab plus chemotherapy vs chemotherapy alone


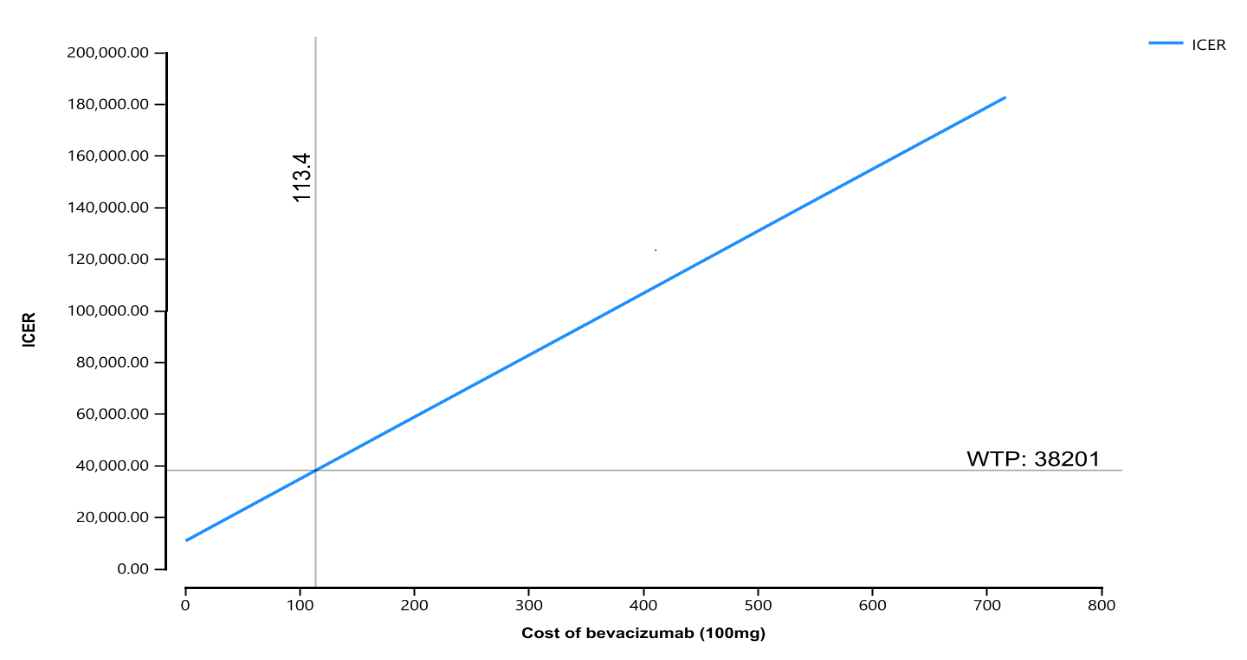


Figure S4. One‐way sensitivity analyses for cost of bevacizumab in scenario analyses
